# Concurrent Granulomatous Mastitis and Ductal Carcinoma In Situ

**DOI:** 10.7759/cureus.38377

**Published:** 2023-05-01

**Authors:** Nana Yoshida, Masaki Nakatsubo, Ryusei Yoshino, Akane Ito, Nanami Ujiie, Sayaka Yuzawa, Masahiro Kitada

**Affiliations:** 1 Thoracic Surgery and Breast Surgery, Asahikawa Medical University Hospital, Asahikawa, JPN; 2 Diagnostic Pathology, Asahikawa Medical University Hospital, Asahikawa, JPN

**Keywords:** breast cancer, cancer, breast, ductal carcinoma in-situ, granulomatous mastitis

## Abstract

Granulomatous mastitis (GM) is a benign inflammatory breast disease that often poses diagnostic challenges due to its similar clinical and radiographic features to breast cancer. We report the case of a 34-year-old female with concurrent GM and ductal carcinoma in situ (DCIS). Initially, breast cancer was suspected based on imaging; however, a needle biopsy confirmed GM. Corticosteroid treatment led to a reduction in tumor size, but subsequent imaging continued to suggest the presence of breast cancer. Surgical excision ultimately revealed the coexistence of GM and DCIS. It is essential to consider the possibility of concurrent breast cancer in cases of GM with discordant imaging and pathology findings.

## Introduction

Granulomatous mastitis (GM) is an inflammatory breast disease characterized by the formation of tumor-like masses, with clinical and radiographic findings often resembling those of breast cancer [1]. There have been cases in which patients were clinically diagnosed with breast cancer and underwent total mastectomy and lymph node dissection, only to receive a subsequent pathological diagnosis of granulomatous mastitis [2]. Conversely, there are rare reports of patients initially diagnosed with GM and treated conservatively who were later found to have breast cancer following surgery due to a lack of improvement [3]. We present an extremely rare case of concurrent GM and ductal carcinoma in situ (DCIS), where breast cancer was initially suspected based on imaging, but a needle biopsy confirmed GM. Following corticosteroid treatment, surgical excision was performed, revealing the coexistence of GM and DCIS.

## Case presentation

**Figure 1:**
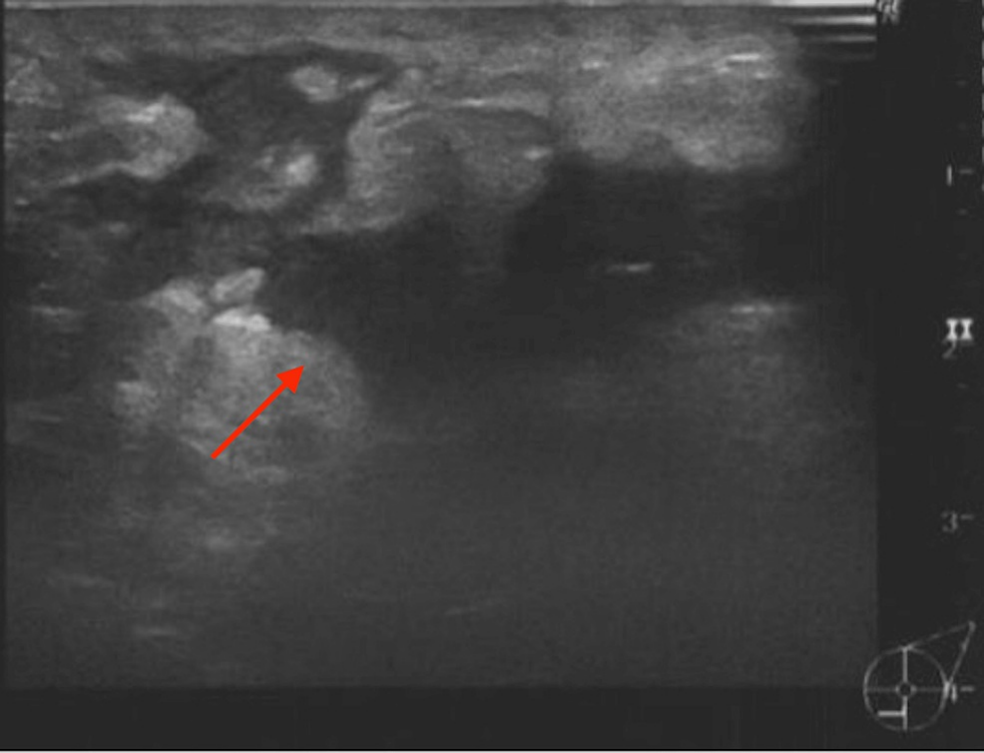
Ultrasonography of the left breast mass. More than 4 cm of the irregular hypoechoic area is observed in the left mammary gland.

We present the case of a 34-year-old female who visited her family doctor with complaints of a mass and redness in her left breast. A fine-needle aspiration cytology was conducted, revealing a mixture of neutrophils, multinucleated histiocytes, and syncytial epithelial cells amidst numerous lymphocytes in sheet-like formations, with no malignant cells detected and benign cellular changes considered. She was subsequently referred to our department for treatment two weeks later. During the initial physical examination, a firm and elastic 4 cm mass in her left breast was palpable, along with some mild redness. The ultrasonography revealed an irregular hypoechoic area of more than 4 cm in the left mammary gland (Figure 1). 

**Figure 2:**
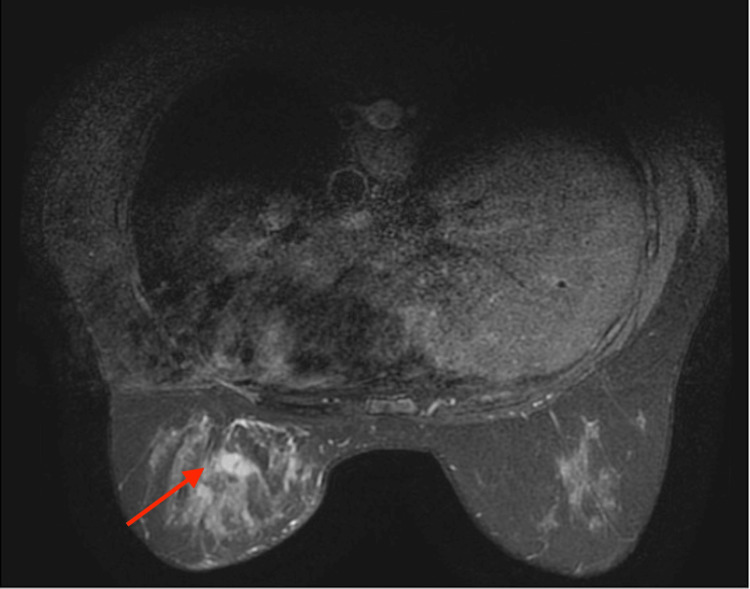
Breast magnetic resonance imaging (T2-weighted imaging). Heterogeneous fibroglandular tissue. Minimal, symmetric background parenchymal enhancement (BPE). Regional non-mass enhancement was noted in the left inner lower part, extending throughout the entire area, as indicated by the arrows. Kinetic curve assessment revealed a fast-washout pattern and pre-spectral edema was also evident. The lesion was classified as breast imaging reporting & data system (BI-RADS) category 4B.

Breast contrast-enhanced magnetic resonance imaging (MRI) showed a widespread T2-weighted imaging high-signal lesion extending to the subcutaneous area in her left breast. The lesion exhibited a fast washout pattern with contrast enhancement, and pre-spectral edema was also observed. No enlarged lymph nodes were detected (Figure 2).

**Figure 3:**
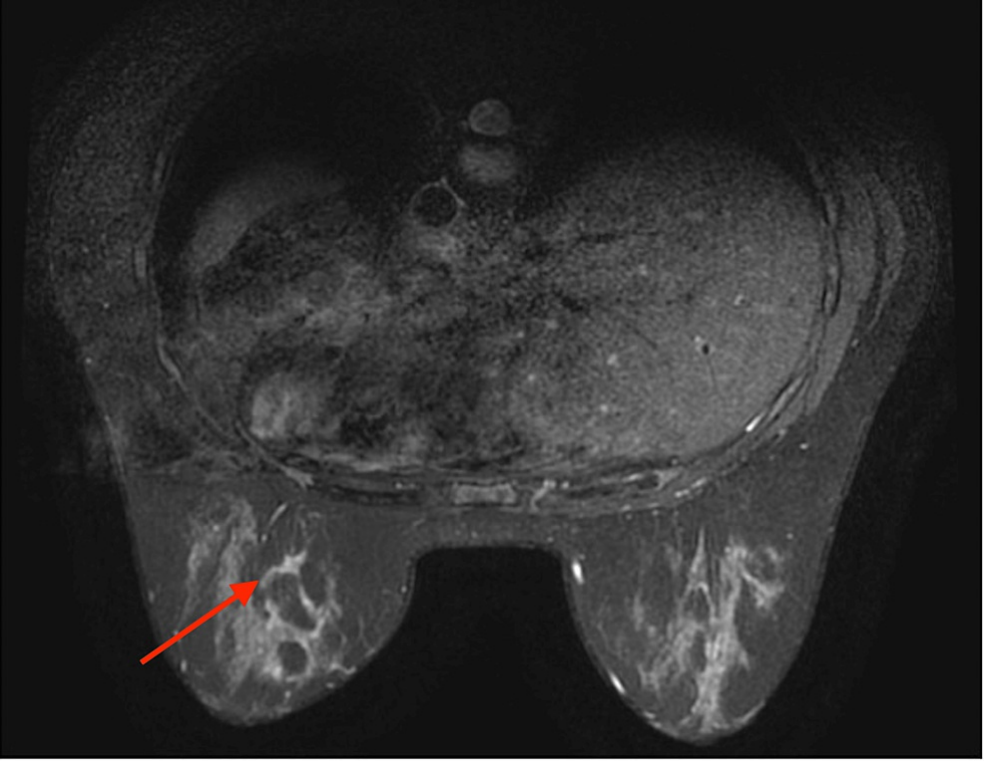
Follow-up breast magnetic resonance imaging (T2-weighted imaging). Regional non-mass enhancement was shrinking but still present as indicated by the arrow. The lesion was classified as breast imaging reporting & data system (BI-RADS) category 4A.

A core biopsy of the left breast was performed. Microscopic examination revealed a significant infiltration of inflammatory cells, including lymphocytes, plasma cells, eosinophils, and neutrophils, within the fibrous tissue. Furthermore, vascular proliferation, fibroblast proliferation, and prominent granuloma formation were observed. No bacteria, fungi, or acid-fast bacilli were detected with Gram, Grocott, and Ziehl-Neelsen staining, respectively. Granulomatous mastitis was considered a potential diagnosis based on these findings. The patient was initially started on oral prednisolone at a dosage of 30 mg/day. After observing tumor shrinkage, the dosage was gradually tapered to 15 mg/day after two weeks and 5 mg/day after four weeks. Nine weeks after the initiation of treatment, the mass exhibited a reduction in size. A follow-up MRI revealed an overall decrease in the extensive T2-weighted high-signal/contrast-enhancing lesion within her left breast. However, small nodular and irregular enhancement effects persisted (Figure 3). 

**Figure 4:**
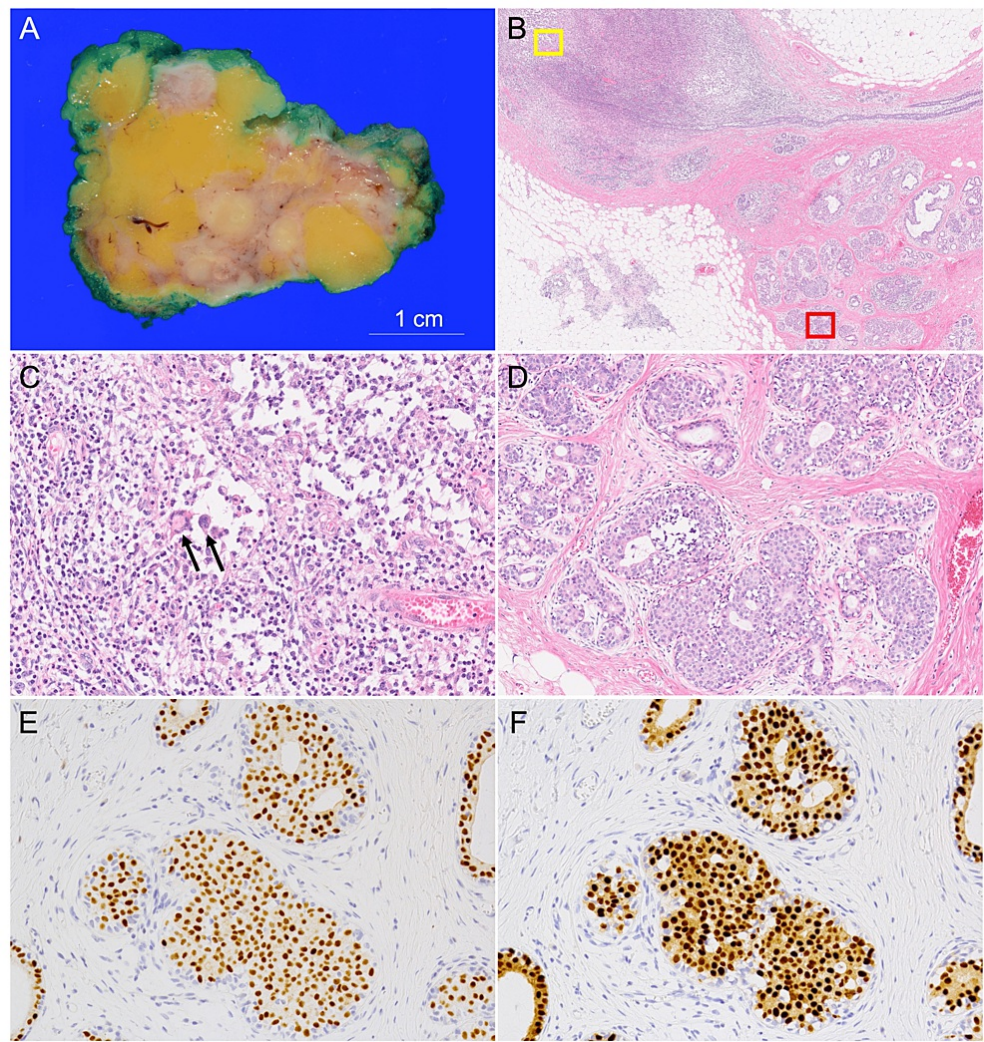
Histopathological findings. (A) Grossly, yellow-white, multi-nodular lesions were observed. (B) Histologically, prominent areas of inflammatory cell infiltration within the lobules (yellow area) and proliferation of the epithelium (red area) were noted. (C) An enlarged view of the yellow region in image (B) showed granulomatous inflammation characterized by the presence of multinucleated giant cells intermingled with neutrophils. (D) An enlarged view of the red region in image (B) showed atypical cells with enlarged, near-circular nuclei, forming a flat, cribriform, or solid pattern within the lobules. (E) The estrogen receptor staining demonstrated a positive result. (F) The progesterone receptor staining demonstrated a positive result.

After a thorough discussion with the patient, it was agreed upon to address the residual mass persisting in the left inframammary region, which corresponds to the area of contrast for the mass on MRI, as illustrated in Figure 3. The resected specimen measured 6.8 × 4 × 4 cm and exhibited multiple yellow-white nodular lesions scattered throughout the cut surface (Figure 4). 

**Figure 5:**
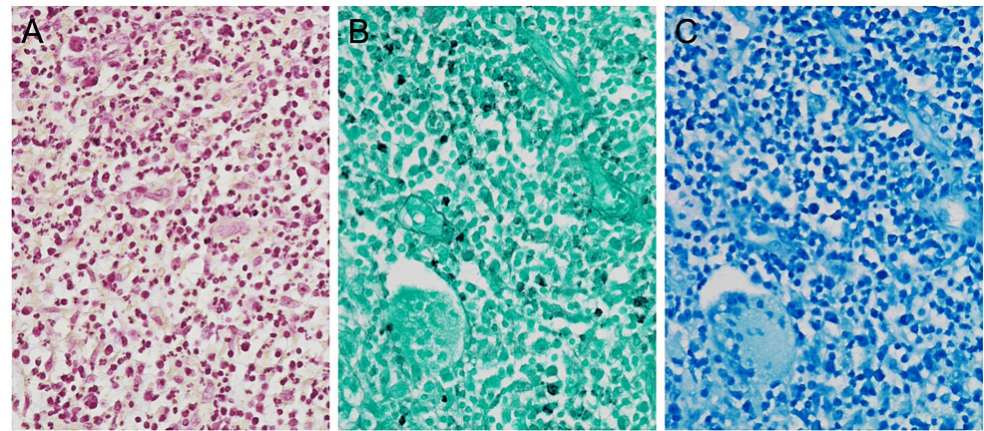
Histopathological findings. (A) Gram staining did not confirm bacteria. (B) Grocott staining did not confirm fungi. (C) Ziehl-Neelsen staining did not confirm *Mycobacterium tuberculosis*.

Histopathological examination performed on a patient's tissue sample. Inflammatory cell infiltration, including lymphocytes, neutrophils, histiocytes, and plasma cells, was observed in multiple nodules. Internal abscess formation, non-caseating epithelioid cell granulomas, and multinucleated giant cells were also seen. No bacteria, fungi, or acid-fast bacilli were detected with Gram, Grocott, and Ziehl-Neelsen staining (Figure 5).

These findings were consistent with granulomatous mastitis, and the lesion was found to be exposed at the margin. In addition, columnar cell lesions, flat epithelial atypia, and atypical ductal hyperplasia (ADH) were observed in the breast tissue, forming a low-grade breast neoplasia pathway. Some areas exhibited atypical cells with enlarged, near-circular nuclei filling the ducts and lobules, exceeding a range of 2 mm, consistent with a low-nuclear-grade DCIS. ADH/DCIS was also observed at the margin. Immunohistochemical analysis revealed that the lesion was positive for estrogen receptor, progesterone receptor, and E-cadherin. The Ki67 labeling index was 10.5%. Due to the positive margin, a decision was made to proceed with a total mastectomy of the left breast. The patient received oral tamoxifen as adjuvant treatment and has maintained a recurrence-free status for 18 months.

## Discussion

Granulomatous mastitis is typically found in women of childbearing age within five years following their last childbirth. Epithelioid cells and neutrophils infiltration, as well as granulomas containing foreign bodies or Langhans giant cells, are histopathological indicators of it. Additionally, the condition often involves abscess formation, primarily affects the lobules, and does not present with caseating granulomas, acid-fast bacteria, or fungi [4]. The etiology of granulomatous mastitis remains uncertain but is thought to be related to factors such as oral contraceptive use, hormonal imbalances including hyperprolactinemia, autoimmune diseases, *Corynebacterium* infections, and trauma [5].

In this case report, we describe a patient presenting with clinical and imaging features of granulomatous mastitis that closely resemble those of breast cancer, posing a diagnostic challenge. The most common clinical presentation is a unilateral mass characterized by indistinct borders. Pain is variable, sometimes present and other times absent. Additional clinical manifestations include erythema, ulceration, and nipple retraction. Mammography frequently revealed asymmetric shadows, succeeded by masses that commonly exhibited irregular boundaries. Ultrasonography demonstrated irregularly shaped hypoechoic masses as the most common observation. MRI depicted granulomatous mastitis as non-mass enhancement (NME), with a clustered ring pattern frequently observed [6]. Differentiating between breast cancer and granulomatous mastitis can be challenging, but it is crucial due to the significant differences in treatment strategies. Currently, histopathological diagnosis is the only reliable method.

While no established guidelines for treatment exist, steroid therapy is often the first choice. There is no consensus on dosage and duration; however, a study comparing high-dose (50 mg/day) tapering treatment with low-dose (5 mg/day) maintenance treatment found significantly higher remission rates and lower relapse rates in the high-dose group [7]. It has been reported that supplementing methotrexate (10-15 mg/week) proves efficacious for patients exhibiting resistance to corticosteroid treatment and experiencing adverse effects such as impaired glucose tolerance and Cushing's syndrome. Furthermore, studies indicate that incorporating methotrexate diminishes the corticosteroid dosage and subsequently mitigates the occurrence of side effects [8]. Recently, it has been documented that steroid injections offer comparable effectiveness, accelerated response time, and reduced recurrence rates compared to steroid-based oral treatments while minimizing the risk of systemic adverse effects [9].

We report an extremely rare case of a patient diagnosed with granulomatous mastitis via needle biopsy who underwent treatment with prednisolone and experienced tumor shrinkage. However, upon re-evaluation with a breast MRI, the possibility of breast cancer could not be entirely excluded, leading to the decision to perform surgical excision. Pathological examination revealed the coexistence of non-invasive ductal carcinoma and granulomatous mastitis, enabling early detection and treatment of the condition.

It is possible that breast cancer and granulomatous mastitis coexisted incidentally in this case. However, considering the patient's young age and lack of family history, along with the intermingled distribution of breast cancer and granulomatous mastitis in the histopathological images, a potential relationship between the two conditions cannot be disregarded. In this report, we suggest that mastitis may be considered a precancerous lesion, but this notion remains unclear. Concurrent GM and DCIS are extremely rare. There are only a few case reports [10-13], and further accumulation of cases is necessary for clarification. The primary message of this paper is that in cases where there is a discordance between imaging and pathological diagnosis, breast cancer might be hidden, and considering surgical resection could be one of the potential solutions.

## Conclusions

The coexistence of GM and breast cancer is an uncommon occurrence, but it warrants consideration when imaging and pathology results don't align. Prompt identification and assertive surgical removal might be required for the best possible outcomes. Additional research is essential to shed light on the connection between GM and breast cancer.
